# From exclusion to deviance: understanding the impact of workplace ostracism on nurses’ deviant behaviors through emotional exhaustion and defensive silence

**DOI:** 10.1186/s12912-025-03427-9

**Published:** 2025-07-01

**Authors:** Nora Mahdy Attia, Manal Saleh Moustafa Saleh, Sahar Hamdy El-Sayed, Marwa Abd El-fatah Ali El-slamoni, Mona Gamal Abd Elnaser Ahmed Elnabawy, Azza Abdeldayem Ata, Abdelaziz Hendy, Enas M. Bassuni, Hanan Meslhy Mohamed

**Affiliations:** 1 Nursing Administration, Faculty of Nursing, Zagazig University, Zagazig, Egypt; 2 Department of Nursing Sciences, College of Applied Medical Science, Shaqra University, Shaqra, Saudi Arabia; 3 Nursing Administration Department, College of Nursing, King Khalid University, Abha, Saudi Arabia; 4 Psychiatric and Mental Health Nursing, Faculty of Nursing, Zagazig University, Zagazig, Egypt; 5 College of Nursing, Taif University, Taif, Saudi Arabia; 6 Nursing Department, College of Applied Medical Science, Taibah University, Yanbu, Saudi Arabia; 7 Pediatric Nursing, Faculty of Nursing, Ain Shams University, Ain Shams, Egypt

**Keywords:** Emotional exhaustion, Defensive silence, Workplace ostracism, Deviant work behaviors

## Abstract

**Background:**

The workplace ostracism phenomenon is gaining more attention and adversely affects organizational outcomes, individual behaviors, and performance. It can directly lead to deviant work behaviors as a retaliatory response. Also, perceived workplace ostracism can lead to emotional exhaustion that can mediate between ostracism and other outcomes. Nurses experiencing emotional exhaustion might adopt defensive silence as a coping strategy, which can contribute to a cycle where unresolved issues and unspoken concerns lead to frustration and disengagement, potentially culminating in deviant work behaviors.

**Aim:**

This study aimed to examine the association between workplace ostracism and nurses’ deviant work behaviors, highlighting the mediating roles of emotional exhaustion and defensive silence in this relationship.

**Subjects and methods:**

A descriptive correlational design was used to describe and examine relationships among these variables in a governmental hospital (Al-Ahrar Teaching Hospitals) in Egypt. Four standardized scales were used to assess workplace ostracism, deviant work behaviors, emotional exhaustion, and defensive silence among nurses; 257 nurses were surveyed randomly. AMOS structural equation modeling (SEM) was used to examine the hypothetical model of the study.

**Results:**

Workplace ostracism had a significant direct effect on emotional exhaustion (β = 0.57, *p* < 0.001), defensive silence (β = 0.47, *p* < 0.001), and deviant work behavior among nurses (β = 0.41, *p* < 0.001). The indirect effect of workplace ostracism on nurses’ deviant work behavior, mediated by emotional exhaustion (β = 0.627, *p* = 0.001). Correspondingly, the indirect effect of workplace ostracism on nurses’ deviant work behavior; mediated by defensive silence (β = 0.033, *p* = 0.041). Also, the overall indirect effect of workplace ostracism on nurses’ deviant work behavior; mediated serially by emotional exhaustion and defensive silence (β = 0.660, *p* = 0.001).

**Conclusions:**

The findings demonstrate that workplace ostracism influences deviant behavior among nurses both directly and indirectly through emotional exhaustion and defensive silence. These results highlight the crucial role of social interaction and trust in minimizing negative workplace behaviors within healthcare settings.

**Implications for nursing management and leadership:**

Addressing workplace ostracism is essential for nursing management to mitigate its negative impact on nurses’ well-being and organizational outcomes. To effectively tackle this issue, nursing managers can implement the following actionable strategies, such as​ creating clear policies that define workplace ostracism and outline procedures for reporting and addressing such behaviors.​ Set up anonymous channels for nurses to report incidents of ostracism without fear of retaliation.​ Regularly monitor and address reports to demonstrate a commitment to a supportive workplace culture.​ Offer workshops focused on communication, empathy, and teamwork to enhance staff interactions and reduce misunderstandings.​ Encourage regular team meetings where nurses can voice concerns, share ideas, and discuss challenges.​ Foster a culture where feedback is welcomed and valued, reducing the likelihood of defensive silence.​ Assess and adjust workloads to ensure they are manageable, thereby reducing stress and emotional exhaustion among staff.​ Acknowledge and reward behaviors that contribute to a positive and inclusive work environment. Celebrate teamwork and collaborative achievements to reinforce a sense of belonging. Implement surveys and assessments to gauge the prevalence of ostracism and its impact on staff.​.

**Clinical trial:**

Not applicable.

## Introduction

Healthcare organizations generate a fostering emergent concern for different healthcare staff, particularly nurses’ attitudes and performance [1]. Governmental hospitals, as a prominent social context, provide nurses with opportunities to engage and converse with other healthcare professionals and patients. While social interactions offer numerous benefits, they are not always positive; some personnel may intentionally be isolated. This phenomenon is known as ostracism, which refers to the extent to which a nurse feels ignored or neglected by others [2]. Workplace ostracism can lead to a range of negative consequences, including decreased job satisfaction, withdrawal of pro-social behaviors, emotional exhaustion, diminished organizational citizenship behaviors, and an increase in deviant behaviors [3].

Deviant workplace behaviors refer to any behavior that violates the social culture, law, values, regulations, traditions, conventions, or customs. These behaviors can take many forms, including dysfunctional behavior, inefficient work practices, organizational misbehaviors, and antisocial behavior [4]. In this study, emotional exhaustion is defined as the tired feeling that arises when emotional resources are drained, preventing nurses from providing nursing care to patients. Additionally, emotional exhaustion is described as a loss of vitality and the feeling that an individual’s emotional resources have been exhausted [5].

Ostracized personnel who perceive a lack of respect from their system or company may use avoidance coping strategies and refuse to communicate tough truths, unfavorable feedback, or opposing viewpoints. They may choose to maintain defensive silence to avoid incurring selfish losses. Defensive silence is a fear-based coping style that involves suppressing relevant thoughts, information, or opinions as a kind of self-protection. The option to participate in this coping strategy may be a cultural artifact, which should insulate personnel from external uncertainties and threats [6]. Previous research studies have found that workplace ostracism increases deviant work behaviors among organizational employees, but these deviances from organizational norms are caused by employees’ emotional exhaustion [7] defensive silence behavior [6], burnout, organizational identification, and organization-based self-esteem [8], psychological needs and the moderating role of perceived inclusive climate [9].

Nurses in Egypt often work in highly demanding environments characterized by chronic shortages of resources, elevated patient-to-nurse ratios, and rigid hierarchical organizational structures. Reports indicate that the average patient-to-nurse ratio in some departments of public hospitals in Egypt may exceed 20:1, compared to international standards that recommend ratios no higher than 6:1 in general care units and 2:1 in intensive care units [10]. Additionally, studies have shown that nearly 40% of nurses in Egypt reported experiencing forms of marginalization or social isolation at work, while another study found that rates of professional burnout exceed 60% among nurses in public hospitals [11, 12].

These systemic challenges increase the likelihood of workplace ostracism, which can, in turn, lead to emotional exhaustion, defensive silence, and ultimately, deviant behaviors in the workplace. Additionally, prevailing cultural norms in Egyptian society often discourage open expression and direct communication, potentially exacerbating feelings of exclusion and allowing unresolved workplace issues to accumulate. Despite the serious implications of these dynamics for staff well-being and the quality of healthcare delivery, there remains a clear lack of empirical research that explores the manifestations and consequences of ostracism in Egyptian healthcare settings.

Therefore, this study represents a pioneering effort to examine how workplace ostracism contributes to deviant behavior among nurses through the mediating roles of emotional exhaustion and defensive silence. By addressing this research gap, the study aims to support the development of culturally sensitive interventions that enhance nurse well-being, improve healthcare outcomes, and reduce deviant behaviors in Egypt’s healthcare sector.

### Literature review and hypothesis development

Ostracism is considered a severe workplace stressor that negatively affects employees’ psychological well-being and work performance. This phenomenon includes avoidance of speech and eye contact, a lack of necessary information, and indifference. Ostracism is defined as the extent to which nurses think that they are excluded or neglected by others [13]. Therefore, when nurses face ostracism, they may feel alienated and undervalued, leading to negative emotions such as frustration and resentment [14]. These negative emotions can, in turn, result in counterproductive workplace behaviors, such as deviant work behavior, which includes actions that violate organizational norms and harm coworkers or the organization itself [15].

According to social exchange theory, when employees perceive a lack of fairness and reciprocity in workplace relationships, they may retaliate through deviant behaviors to restore a sense of balance. Nurses who experience ostracism may withdraw from their professional responsibilities, show reduced commitment, or even engage in behaviors that negatively impact patient care [16]. Previous research suggests that ostracized employees often engage in interpersonal and organizational deviance as a way to cope with exclusion [17]. Based on these findings, the following hypothesis is proposed:

**H1: **Workplace ostracism is positively associated with deviant work behavior among nurses.

Workplace deviance can take the form of tardiness, stealing, absenteeism, and verbal abuse, which directly affects an organization, resulting in lower productivity and commitment, and greater employee turnover [18]. Nurses who encounter such deviant behaviors at the workplace are more likely to have lower job motivation, quit, and feel stress on the job, which will eventually lead to low self-esteem, psychiatric disorders, and greater fear of downsizings [19].

The Job Demands-Resources (JD-R) model posits that excessive workplace demands such as ostracism deplete nurses’ emotional and psychological resources, leading to emotional exhaustion and deviant work behaviors [20]. Emotional exhaustion is a prolonged state of emotional and physical depletion caused by high professional and/or personal obligations, as well as ongoing stress [21]. Emotional weariness in nurses is associated with medication errors, infections, poor teamwork, anxiety, job dissatisfaction, absenteeism, turnover, mental health issues, and reduced job performance, ultimately compromising patient care and safety. Consequently, this phenomenon should be exacerbated in healthcare professionals by strong leadership, nurses’ involvement in decision-making, increased autonomy, positive nurse-physician relationships, and proper staffing [22, 23].

Several studies have demonstrated that workplace ostracism contributes to emotional exhaustion because employees who feel socially excluded lack the necessary social support to buffer workplace stress. When nurses experience ostracism, they may struggle with feelings of helplessness and emotional fatigue, making it difficult to remain engaged and motivated at work. As a result, ostracized nurses may become emotionally drained, leading to further negative work outcomes [24]. Consequently, the following hypothesis was proposed:

**H2: **Workplace ostracism is positively associated with emotional exhaustion among nurses.

Nurses who experience workplace ostracism may perceive that their input is neither valued nor welcomed, making them more likely to remain silent to avoid further exclusion or punishment [25]. Accordingly, when nurses experience emotional exhaustion, they may lack the psychological and emotional resources needed to actively engage in workplace communication and decision-making, leading to defensive silence that is a self-protective behavior in which nurses deliberately withhold their opinions, concerns, or ideas out of fear of negative consequences [26]. Healthcare is regarded as a workplace that requires healthcare professionals’ voices. Although speaking up is vital for improving patient safety, some scholars have noted that healthcare personnel frequently remain silent, consequently reinforcing the feeling of being ignored or excluded [27]. Organizational silence was initially defined as an individual’s decision to withhold their opinions, ideas, and information on the potential problems within the organization [28].

When nurses struggle to express their thoughts over time, it negatively affects their mental and physical well-being. As well, organizational silence obstructs problem detection, learning, and innovation in healthcare settings [29]. This phenomenon harms job satisfaction, performance, patient safety, as well as employees’ and organizational development due to a lack of individual participation in organizational concerns. Even though nurses’ opinions on patients’ safety may help prevent patients’ mishaps [30, 31]. When silence happens, nurses recognize a lack of control, feel useless, or experience cognitive conflict [32]. Additionally, research indicates that when employees feel excluded, they avoid engaging in constructive discussions or raising concerns, leading to communication breakdowns and potential workplace inefficiencies [28]. Therefore, the following hypothesis was suggested:

**H3: **Workplace ostracism is positively associated with defensive silence among nurses.

Because nursing is a high-pressure and emotionally demanding profession, nurses are especially susceptible to the negative impacts of workplace ostracism. One of the primary psychological mechanisms through which workplace ostracism influences behavior is emotional exhaustion, a core component of burnout that reflects chronic workplace stress [33]. Emotional exhaustion, in turn, may lead to deviant work behavior, which includes acts of aggression, withdrawal, or counterproductive workplace behaviors [34]. Additionally, the Conservation of Resources (COR) theory posits that nurses strive to acquire, maintain, and protect valuable resources such as energy, social support, and self-esteem. When nurses experience workplace ostracism, they perceive a loss of critical social and emotional resources by making them feel excluded and undervalued, leading to emotional exhaustion. In response to this resource depletion, nurses who lack adequate coping mechanisms may resort to deviant work behaviors as a way to manage their stress and frustration [35]. Accordingly, we put forward the following hypothesis:

**H4:** Emotional exhaustion mediates the relationship between workplace ostracism and deviant work behavior among nurses.

In nursing, where teamwork and communication are critical for patient care, ostracism can create a toxic work environment, leading to various negative consequences, including increased defensive silence and deviant work behaviors [36]. Defensive silence, characterized by employees withholding information or concerns due to fear of negative consequences, may act as a psychological coping strategy for ostracized nurses [26]. However, this silence can further contribute to workplace dysfunction, ultimately leading to deviant work behaviors such as withdrawal, reduced cooperation, or even retaliation [37]. Furthermore, the fear-avoidance model suggests that employees remain silent due to fear of negative consequences. Consequently, ostracized nurses may fear further exclusion or punishment if they speak up, leading them to engage in defensive silence, which suppresses constructive communication and fosters disengagement, ultimately contributing to deviant behaviors [38]. Subsequently, we proposed the following hypothesis:

**H5: **Defensive silence mediates the relationship between workplace ostracism and deviant work behaviors among nurses.

Workplace ostracism is a problematic social feature in personnel who experience ostracism are deprived of social support and may feel isolated and undervalued, leading to emotional exhaustion [39]. In such a situation, most employees become mute observers and adopt defensive silence as a self-protection policy. By withholding their ideas or concerns, they aim to protect themselves from further emotional depletion and avoid conflicts that could exacerbate their exhaustion [32]. Defensive silence can contribute to a cycle where unresolved issues and unspoken concerns lead to frustration and disengagement, potentially culminating in deviant work behaviors [40]. Nurses who experience ostracism may feel that their contributions are undervalued. In response, they first experience emotional exhaustion, then adopt defensive silence as a protective strategy, and finally engage in deviant behaviors as a form of perceived retaliation against the organization or colleagues [41]. As a result, we put forward the following hypothesis.

**H6: **Emotional exhaustion and defensive silence serially mediate the relationship between workplace ostracism and deviant work behavior*.*

**Fig. 1:**
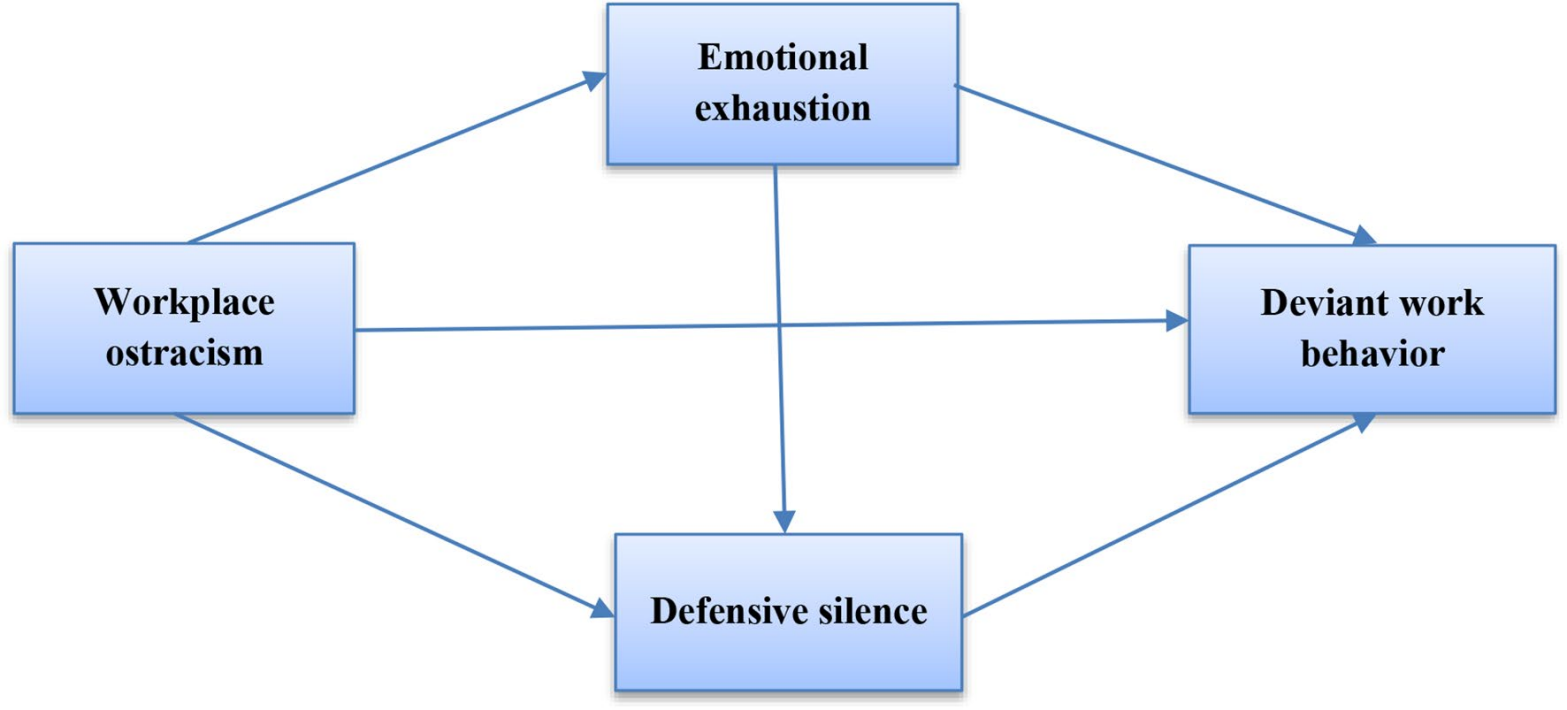
The conceptual model of the study

Taking into account the six hypotheses mentioned above, the conceptual research model is presented as follows (Fig. 1):

### The study objective

This study aimed to examine the association between workplace ostracism and nurses’ deviant work behaviors, highlighting the mediating roles of emotional exhaustion and defensive silence in this relationship.

## Methodology

### Study design

A descriptive-correlational design was chosen. This design combines descriptive and correlational methods to describe population characteristics and examine relationships between variables, assessing factors’ relationships and identifying patterns or trends [42].

### Study setting

This study was carried out in a governmental hospital (Al-Ahrar Teaching Hospitals) in Zagazig City in Egypt that offers comprehensive medical services. The hospital plays a vital role in advancing Egypt’s public healthcare system through its integrated approach to service, training, and research.

### Participants

An open-source sample size calculator was utilized to determine the necessary sample size based on the total population of the previous hospital. The formula used was n = [N/ 1 + N (e)^2^] [43], where N is the total population size and n is the sample size. The calculations were made with a confidence level of 95%, a margin of error of 5.0%, and a total population size of 500 staff nurses. The sample size was calculated to be 223 staff nurses. After accounting for a 15% dropout rate (34 staff nurses), the required sample size was adjusted to include 257 licensed staff nurses who were on duty during the study period and who had at least one year of experience at their current hospital to ensure familiarity with workplace dynamics.

**Fig. 2:**
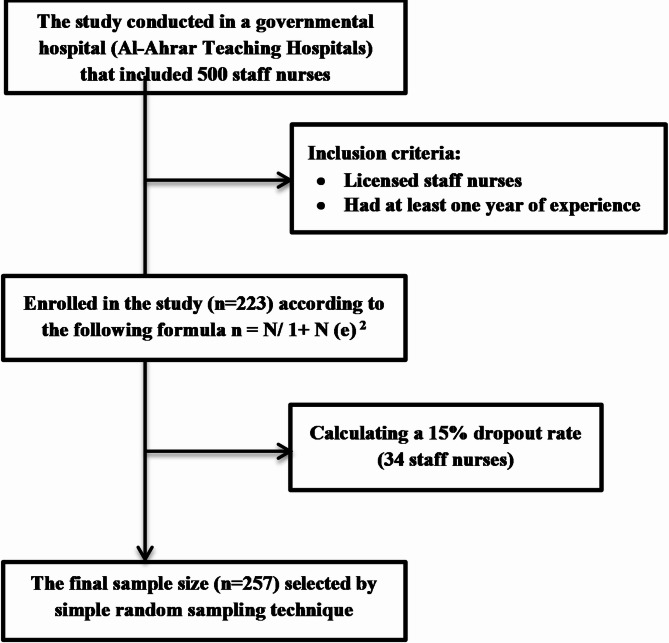
Flow diagram of the sampling process

Once the researchers obtained the required number of nurses, a simple random sampling technique was used to select participants, in which the researchers created a complete list of all nurses in the hospital. Then they assigned a unique number to each nurse in the sampling frame. Following that they wrote nurses’ numbers on slips of paper, placed them in a box, mixed them thoroughly, and picked numbers randomly until the corresponding nurses were included in the sample (Fig. 2**)**.

### Instruments used in the study

The data for this study were obtained using four standardized scales.

#### Workplace ostracism scale

This standardized scale was developed by Ferris et al. [44] and modified by El-Guindy et al. [45] to assess the degree of workplace ostracism perceived by nurses. The 20 items were split into two categories as follows: the perception of exclusion (10 items, e.g., others ignored you at work) and the personal impact of exclusion (10 items, e.g., being ignored by others at work has a personal impact on you). Every item on the scale was rated using a five-point Likert scale. The scale went from 1 = strongly disagree to 5 = strongly agree. The participants’ responses were summed up and then divided by the number of items to calculate a mean score. The total score of the workplace ostracism scale ranged from 1 to 5. The scoring system for the tool is determined by specific cutoff points: A score above 3.75 (˃ 75%) signifies a high level of workplace ostracism, a score ranging from 2.5 to 3.75 (50–75%) indicates a moderate level, and a score below 2.5 (˂ 50%) represents a low level.

Factor analysis was conducted to assess the questionnaire’s validity, while Cronbach’s alpha coefficient was utilized to measure its reliability. In this study, the internal consistency reliability of the scale was assessed using the Cronbach’s alpha coefficient, which was found to be 0.842, confirming its reliability. The Cronbach’s α test for the subscales was as follows: Perception of exclusion (0.930) and personal impact of exclusion (0.908). The Kaiser-Meyer-Olkin (KMO) measure of sampling adequacy was 0.932, suggesting that the data were suitable for factor analysis. Additionally, Bartlett’s test of sphericity was statistically significant (*P* = 0.000), validating the factorability of the correlation matrix. Consequently, all scale items were retained.

#### Emotional exhaustion scale (EES)

Emotional exhaustion was measured using the open-access nine-item Emotional Exhaustion Scale created by Maslach and Jackson [46], e.g., I feel emotionally drained from my work. Each item on the scale was rated using a seven-point Likert scale, ranging from 0 (never) to 6 (every day). The participants’ responses were summed and then divided by the number of items to calculate a mean score. The overall score for the emotional exhumation scale ranged from 1 to 5. The tool classifies scores based on specific cutoff points: Emotional exhaustion is considered high if the score exceeds 3.75 (˃ 75%); moderate if it falls between 2.5 and 3.75 (50–75%); and low if it is below 2.5 (˂ 50%).

Factor analysis was performed to evaluate the questionnaire’s validity, while Cronbach’s alpha coefficient was used to assess its reliability. This study measured the scale’s internal consistency using Cronbach’s alpha, which was 0.888, confirming its strong reliability. The Kaiser-Meyer-Olkin (KMO) measure of sampling adequacy was 0.945, indicating that the data were appropriate for factor analysis. Furthermore, Bartlett’s test of sphericity was statistically significant (*P* = 0.000), supporting the factorability of the correlation matrix. As a result, all scale items were retained. An official to use the Emotional Exhaustion Scale from Mind Garden, Inc.: Order #75,067.

#### Defensive silence scale

This standardized scale was developed by Çakıcı [47] and used in Eriguc et al. [48] to identify the issues on which nurses remain silent. This scale consisted of 31 items. These items were divided into five dimensions: organizational position (2 items e.g. having a low position), fears about the work (6 items e.g. fear of lack of promotion), lack of experience (3 items e.g. the concern that ignorance and inexperience are noticed), isolation and fear of relationship damage (8 items e.g. fear of the loss of support), and administrative and organizational reasons (12 items e.g. mistrust towards the administrators). The nurses’ responses were rated on a five-point Likert scale, with 5 representing “strongly agree” and 1 representing “strongly disagree.” The participants’ replies were summed and divided by the number of items to compute the mean score. The defensive silence scale ranges from 1 to 5, with scores classified based on predefined cutoff points: a score above 3.75 (˃ 75%) indicates a high level of defensive silence, a score between 2.5 and 3.75 (50–75%) represents a moderate level, and a score below 2.5 (˂ 50%) signifies a low level.

The questionnaire’s validity was evaluated by factor analysis, and its reliability was assessed using Cronbach’s alpha coefficient. Cronbach’s alpha was used in this study to determine the scale’s internal consistency, which was 0.960, demonstrating its high reliability. The Cronbach’s α reliability coefficient for the subscales was as follows: Organizational position (0.850), fear of work (0.832), lack of experience (0.825), isolation and fear of destroying relationships (0.920), and administrative and organizational reasons (0.912). The Kaiser-Meyer-Olkin (KMO) index of sample adequacy was 0.932, indicating that the data were suitable for factor analysis. Additionally, Bartlett’s sphericity test was statistically significant (*P* = 0.000), indicating that the correlation matrix is factorable. As a result, all scale items were kept.

#### Workplace deviance questionnaire (WDQ)

This standardized WDQ was developed by Bennett and Robinson [34] to measure the frequency with which nurses engage in detrimental behaviors toward other employees or the organization. The 19 items were grouped into two dimensions: Interpersonal deviance (7 items, e.g., acted rudely toward someone at work) and organizational deviance (12 items, e.g., took property from work without permission). Every item on the scale was rated using a five-point Likert scale ranging from 1 = never to 5 = daily. The participants’ responses were summed up and then divided by the number of items to calculate a mean score. The total score of the workplace deviant behavior questionnaire ranges from 1 to 5 and is categorized based on cutoff points: scores above 3.75 (˃ 75%) indicate a high level of deviant behavior, scores between 2.5 and 3.75 (50–75%) reflect a moderate level, and scores below 2.5 (˂ 50%) signify a low level.

Factor analysis was conducted to assess the questionnaire’s validity, while Cronbach’s alpha coefficient was utilized to measure its reliability. The internal consistency reliability of the scale was assessed using the Cronbach’s alpha coefficient, which was found to be 0.937, confirming its reliability. The Cronbach’s α reliability coefficient for the subscales were as follows: Interpersonal deviance (0.870) and organizational deviance (0.893). The Kaiser-Meyer-Olkin (KMO) measure of sampling adequacy was 0.904, suggesting that the data was suitable for factor analysis. Additionally, Bartlett’s test of sphericity was statistically significant (*P* = 0.000), validating the factorability of the correlation matrix. Consequently, all scale items were retained.

#### Demographic information of study participants

Participants’ Demographic data were collected, including gender, age, job position, level of education, and years of experience.

### Translation and validity of the tools

To guarantee that the participating nurses, whose first language is Arabic, fully comprehended the study measures, all items were translated into Arabic. A panel of five university professors proficient in both Arabic and English reviewed the translation. Additionally, a second panel of five bilingual university professors conducted a back-translation from Arabic to English. Seven nursing professors specializing in nursing administration thoroughly evaluated the measures to assess content and face validity. The panelists confirmed the measures’ validity for this study, determining that no modifications were necessary based on their recommendations.

### Pilot study

A pilot study was conducted to evaluate the quality and clarity of the intervention materials, the time required for data collection, the feasibility, validity, and reliability of the study measurements. The pilot study involved 26 nurses (10% of the sample size) who met the inclusion criteria but were not part of the study population. The results indicated that no changes were necessary, and the study’s measures were unambiguous. The internal consistency was confirmed with Cronbach’s alpha values ranging from 0.842 to 0.960 for all scale, indicating highly internal consistency.

### Data collection

Staff nurses’ self-reported assessments were used to gather data for this study between March and the end of May 2024. Before any data was collected, the required ethical approvals were obtained. At the first meeting, the chief nurse of each hospital unit was briefed about the goals of the study and asked for assistance in helping to streamline the data collection procedure. Nurses who met the inclusion criteria received sealed envelopes containing the survey questionnaires in order to improve perceived anonymity and minimize potential response bias. Throughout their shifts, these envelopes were distributed to them in their work areas.

The questionnaire’s cover letter made clear why the study was being conducted, assured participants that their answers would be anonymous and voluntary, and gave them instructions to think back on their interactions with a particular nurse manager. Nurses were asked to return their completed questionnaires in sealed envelopes to a designated drop box in a common area within each unit by a specified deadline to further ensure confidentiality and alleviate any pressure. The voluntary completion and submission of the surveys suggested participation. The goal of this approach was to reduce direct communication between the research team and submitters to create a more private and objective setting where sincere answers could be given.

### Ethical considerations and consent to contribute

The Ethics Committee of the Faculty of Nursing at Zagazig University, Egypt, granted ethical approval for this study (Reference number 138 − 24). In the initial section of the informed consent form, participants were provided with all relevant information about the study. Before starting the survey, nurses were informed about the study’s aim and nature, and they were asked to indicate their agreement to provide signed consent, per the criteria outlined in the Helsinki Declaration. Each questionnaire was assigned a unique code number to ensure confidentiality and anonymity. Nurses were assured that the collected data would be used solely for research. The respondents were guaranteed the privacy and confidentiality of their answers, the voluntary nature of their involvement, and the right to withdraw from the study at any time. The informed consent was obtained through questionnaire completion.

### Statistical design

The data were analyzed using AMOS (Analysis of Moment Structures) 26 and IBM SPSS 25. Descriptive statistics were used to summarize the study variables and characteristics of studied nurses. Independent t-tests and analysis of variance (ANOVA) were conducted to examine differences in workplace ostracism, deviant work behavior, emotional exhaustion, and defensive silence based on sample characteristics. Pearson’s correlation analysis was applied to explore the strength and direction of the bivariate relationships between the research variables.

AMOS Structural Equation Modeling (SEM) was employed to test the proposed model, allowing for the examination of complex relationships between variables while accounting for measurement errors. SEM is particularly effective for assessing mediation effects, as it enables simultaneous analysis of both direct and indirect relationships, providing a more comprehensive understanding than traditional regression methods [49]. Additionally, the validity and reliability of the study constructs were assessed. Statistical significance was determined using a two-tailed p-value of < 0.05, while a two-tailed p-value of < 0.01 indicated high statistical significance.

## Results of the study

Table 1 displays applicants’ demographics and variances in the study variables. The applicants were aged from 30 to 40 years old (44.7%). Most of them were female and worked as bedside nurses (92.2% and 72.4%, respectively). Also, 64.2% obtained a bachelor’s nursing degree, and 47.1% spent more than 20 years in the nursing profession. Moreover, there was a statistically significant difference in the study variables according to nurses’ years of experience (*p* < 0.01).

**Table 1:** Participants’ demographics and variances in the study variables (*n* = 257)

Characteristic	Category	No. (%)	Workplace ostracism		Deviant work behavior		Emotional exhaustion		Defensive silence	
			**M (SD)**	**t/F (p)**	**M (SD)**	**t/F (p)**	**M (SD)**	**t/F (p)**	**M (SD)**	t/F (p)
Age (years) ^a^	**< 30**	96 (37.4)	3.05 (0.48)	F = 0.702(0.49)	3.28 (0.72)	F = 0.783 (0.458)	3.25 (0.78)	*F = 1.58 (0.20)*	3.30 (0.70)	*F = 0.92 (0.39)*
	**30–40**	**115 (44.7)**	3.15 (0.50)		3.36 (0.67)		3.36 (0.72)		3.39 (0.67)	
	**> 40**	46 (17.9)	3.12 (0.73)		3.45 (0.97)		3.52 (0.98)		3.49 (0.95)	
Gender ^b^	**Male**	20 (7.8)	3.09 (0.40)	t = 0.205 (0.83)	3.28 (0.13)	t = 0.466 (0.64)	3.27 (0.61)	*t = 0.526 (0.60)*	3.35 (0.53)	*t = 0.144 (0.88)*
	**Female**	**237 (92.2)**	3.11 (0.55)		3.35 (0.76)		3.35 (0.81)		3.37 (0.76)	
Position a	**Staff**	**186 (72.4)**	3.10 (0.52)	F = 0.29(0.74)	3.35 (0.70)	F = 0.08 (0.92)	3.35 (0.75)	*F = 0.41 (0.66)*	3.38 (0.69)	*F = 0.16 (0.84)*
	**Charge**	61 (23.7)	3.08 (0.69)		3.34 (1.00)		3.38 (1.04)		3.32 (1.02)	
	**Supervisors**	10 (3.9)	3.23 (0.38)		3.25 (0.60)		3.13 (0.65)		3.28 (0.57)	
Education ^a^	**Diploma**	28 (10.9)	3.13 (0.52)	F = 0.19 (0.98)	3.29 (0.87)	F = 0373 (0.68)	3.24 (0.93)	*F = 0.41 (0.65)*	3.33 (0.88)	*F = 0.22 (0.80)*
	**Associate**	64 (24.9)	3.10 (0.64)		3.43 (0.86)		3.42 (0.90)		3.43 (0.88)	
	**Bachelor**	**165 (64.2)**	3.10 (0.51)		3.33 (0.69)		3.33 (0.74)		3.36 (0.67)	
years of experience in the hospital (years) ^a^	**< 5**	18 (7.0)	3.56 (0.34)	F = 4. 67 (0.001)*	4.01 (0.61)	F = 4.49 (0.002)*	3.99 (0.62)	F = 3.67 (0.006)*	4.00 (0.61)	F = 4.25 (0.002)*
	**5–10**	36 (14.0)	3.17 (0.53)		3.42 (0.75)		3.43 (0.82)		3.49 (0.79)	
	**10–15**	40 (15.6)	2.87 (0.60)		3.00 (0.89)		3.00 (0.96)		3.03 (0.89)	
	**15–20**	42 (16.3)	3.23 (0.52)		3.42 (0.69)		3.35 (0.76)		3.37 (0.74)	
	> 20	121 (47.1)	3.07 (0.51)		3.33 (0.69)		3.35 (0.73)		3.38 (0.65)	

(M) Mean. (SD) Standard deviation. (a) (F) One-way analysis of variance. (b) *t*-test for the independent group. * *(p) Statistically significant at p* < 0.01

Table 2 presents the distribution of the mean percentage scores for the study variables as reported by the nurses. As shown in the table, workplace ostracism and emotional exhaustion had mean percentage scores of 63% and 67.6%, respectively, indicating moderate levels. Similarly, nurses’ defensive silence and deviant work behaviors showed moderate levels, with mean percentage scores of 67.8% and 67.6%, respectively. Among the dimensions of workplace ostracism, the personal impact of exclusion had the highest mean percentage score at 67.2%, while the perception of exclusion had the lowest at 58.8%. Likewise, fear about the work constituted the highest mean percent score among defensive silence dimensions, while organizational position had the lowest (70.6% and 61.2%, respectively). Similarly, within the dimensions of deviant work behaviors, interpersonal deviance recorded the highest mean percentage score at 69%, whereas organizational deviance had the lowest at 66.6%.

**Table 2:** Distribution of different study variables’ mean percent scores and cronbach’s alphas as reported by the studied nurses (*n* = 257)

Variable	Maximum score		Mean (SD)	Mean percentage scores	α
Workplace ostracism dimensions:					
• Perception of exclusion	5	2.94 (0.42)		**58.8%**	0.930
• Personal impact of exclusion	5	3.36 (0.79)		**67.2%**	0.908
Total workplace ostracism	5	**3.15 (0.54)**		**63%**	0.842
Total emotional exhaustion	5	**3.38 (0.82)**		**67.6%**	0.888
Defensive silence dimensions:					
• Organizational position	5	3.06 (1.06)		**61.2%**	0.850
• Fear of the work	5	3.53 (0.87)		**70.6%**	0.832
• Lack of experience	5	3.31 (0.97)		66.2%	0.825
• Isolation and fear of relationship damage	5	3.39 (0.83)		67.8%	0.920
• Administrative and organizational reasons	5	3.42 (0.82)		68.4%	0.912
Total defensive silence	5	**3.39 (0.78)**		**67.8%**	0.960
Deviant work behaviors dimensions:					
• Interpersonal deviance	5	3.45 (0.82)		**69%**	0.870
• Organizational deviance	5	3.33 (0.78)		**66.6%**	0.893
Total deviant work behavior	5	3.38 (0.78)		67.6%	0.937

SD Standard deviation. α = Cronbach’s alphas

Table 3 examines the correlation coefficients among the study variables. As seen in the table, there were significant and positive associations between workplace ostracism as regards emotional exhaustion (*r* = 0.623, *p* < 0.01), defensive silence (*r* = 0.674, *p* < 0.01), and deviant work behavior (*r* = 0.743, *p* < 0.01). This suggests that workplace ostracism increases emotional exhaustion by draining nurses’ emotional resources, fosters defensive silence as a self-protective response, and promotes deviant behavior due to the stress and frustration that ostracism causes. These findings emphasize the harmful effects of ostracism on employee well-being and behavior.

Similarly, emotional exhaustion was positively related to defensive silence (*r* = 0.818, *p* < 0.01) and deviant work behavior (*r* = 0.862, *p* < 0.01). This indicates that nurses who are emotionally exhausted and approve of the defensive silence are certainly involved in deviant work behaviors. Besides, defensive silence correlated positively with deviant work behavior (*r* = 0.829, *p* < 0.01), signifying that nurses who withhold their opinions their thoughts, concerns, or feedback out of fear or self-protection are more likely to engage in counterproductive workplace behaviors.

Also, the two dimensions of workplace ostracism (perception of exclusion and personal impact of exclusion) were associated positively with nurses’ emotional exhaustion, defensive silence, and their deviant work behavior, where p-value < 0.01. Additionally, all five dimensions of defensive silence organizational position, fear about the work, lack of experience, isolation and fear of relationship damage, and administrative and organizational reasons—showed a positive association with nurses’ workplace ostracism, emotional exhaustion, and deviant work behavior, with a statistically significant p-value of less than 0.01. Furthermore, both dimensions of deviant work behavior, interpersonal deviance and organizational deviance, were positively associated with nurses’ workplace ostracism, emotional exhaustion, and defensive silence, all with a statistically significant p-value of less than 0.01.

**Table 3:** Analyzing the correlation coefficients among the study variables (*n* = 257)

Variable	Perception of exclusion	Personal impact of exclusion	Total workplace ostracism	Emotional exhaustion	Organizational position	Fear about the work	Lack of experience	Isolation and fear of relationship damage	Administrative and organizational reasons	Total defensive silence	Interpersonal deviance	Organizational deviance
Personal impact of exclusion (r)	0.365*											
Total workplace ostracism (r)	0.586*	0.811*										
Emotional exhaustion (r)	0.487*	0.575*	0.623*									
Organizational position (r)	0.473*	0.564*	0.604*	0.738*								
Fear about the work (r)	0.370*	0.568*	0.574*	0.760*	0717*							
Lack of experience (r)	0.253*	0.762*	0.631*	0.465*	0474*	0.465*						
Isolation and fear of relationship damage (r)	0.390*	0.549*	0.556*	0.576*	0.564*	0.548*	0.496*					
Administrative and organizational reasons (r)	0.343*	0.533*	0.519*	0.511*	0.502*	0.482*	0.456*	0640*				
Total defensive silence (r)	0.440*	0.660*	0.674*	0.818*	0814*	0.755*	0.573*	0723*	0.625*			
Interpersonal deviance (r)	0.536*	0.582*	0.656*	0.839*	0.767*	0706*	0404*	0.561*	0.512*	0.729*		
Organizational deviance (r)	0.455*	0.755*	0.750*	0.797*	0.789*	0662*	0.656*	0598*	0565*	0.815*	0.728*	
Total deviant work behaviors (r)	0.497*	0.708*	0.743*	0.862*	0.824*	0.700*	0.563*	0602*	0564*	0.829*	0.838*	0.910*

r = Pearson correlation coefficient, measuring the strength and direction of the relationship between variables. * Statistically significant at *p* < 0.01 (2-tailed)

Note. Strength of correlation interpretation: 0.00-0.10 = Negligible correlation, 0.10–0.39 = Weak, 0.40–0.69 = Moderate, 0.70–0.89 = Strong, and 0.90–1.00 = Very strong

**Fig. 3:**
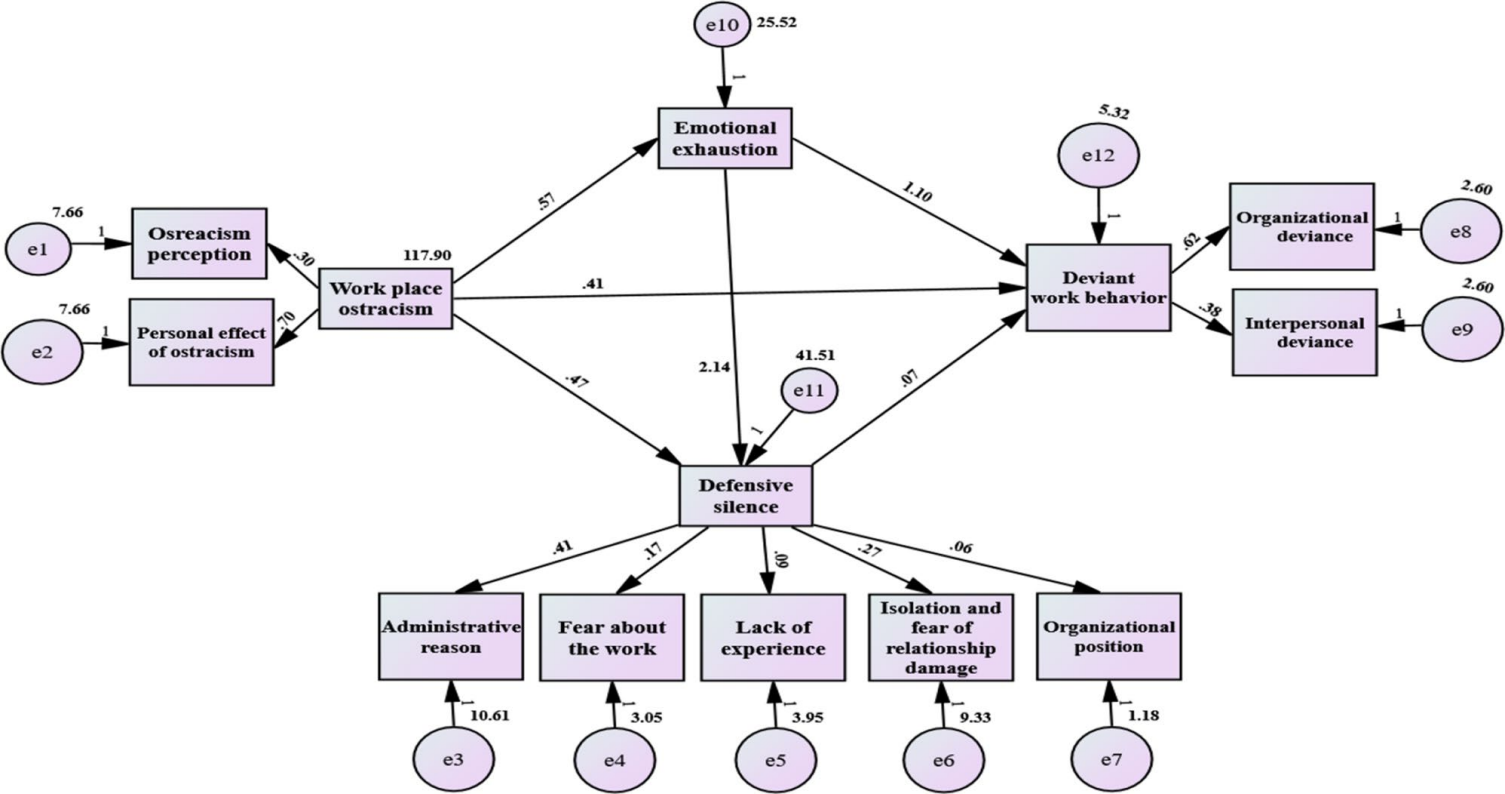
The mediating effects of emotional exhaustion and defensive silence on the relationship between workplace ostracism and deviant work behaviors (*n* = 257)

Table 4 illustrates the direct and indirect effects. Figure 3 pictures the mediating effects of emotional exhaustion and defensive silence on the relationship between workplace ostracism and deviant work behaviors. As displayed, workplace ostracism had a significant direct effect on emotional exhaustion (β = 0.57, *p* < 0.001), defensive silence (β = 0.47, *p* < 0.001), and deviant work behavior among nurses (β = 0.41, *p* < 0.001). Likewise, emotional exhaustion had a significant direct effect on defensive silence (β = 2.14, *p* < 0.001) and deviant work behavior among nurses (β = 1.10, *p* < 0.001). Defensive silence also significantly affected deviant work behavior (β = 0.07, *p* < 0.041).

The indirect effect of workplace ostracism on nurses’ deviant work behavior, mediated by emotional exhaustion, is calculated by multiplying the coefficients of the direct effects: the effect of workplace ostracism on emotional exhaustion and the effect of emotional exhaustion on deviant work behavior (0.57 × 1.10 = 0.627, *p* = 0.001, 95% CI [0.503, 0.739]), representing that the relationship between workplace ostracism and deviant work behavior among staff nurses was mediated by emotional exhaustion. This means that for each 1-unit increase in workplace ostracism, nurses’ deviant work behavior is expected to increase by 0.627 units indirectly through the effect of emotional exhaustion.

Correspondingly, the indirect effect of nurses’ workplace ostracism on their deviant work behavior through the mediator—defensive silence is determined by multiplying the coefficients of both direct effects: the effect of workplace ostracism on defensive silence and the effect of defensive silence on deviant work behavior (0.47 × 0.07 = 0.033, *p* = 0.041, 95% CI [0.001/0.098]), demonstrating that defensive silence acted as a mediating factor in the relationship between workplace ostracism and deviant work behavior among staff nurses. This indicates that a 1-unit increase in workplace ostracism is associated with a 0.033-unit rise in deviant work behavior through the influence of defensive silence.

Likewise, the overall indirect effect of workplace ostracism on nurses’ deviant work behavior, mediated by emotional exhaustion and defensive silence, is calculated by summing the coefficients of both indirect pathways (0. 0.627 + 0.033 = 0.660, *p* = 0.001, 95% CI [0.516/0.788]), signifying that these two factors serially mediate the relationship between workplace ostracism and deviant work behavior among staff nurses. Consequently, for every 1-unit increase in workplace ostracism, there is an expected 0.660 unit increase in deviant work behavior, occurring indirectly through the combined effects of emotional exhaustion and defensive silence. Overall, these findings highlight the complex pathways through which workplace ostracism contributes to deviant work behavior and emphasize the need for supportive workplace environments to mitigate emotional exhaustion and promote open communication.

To ensure the goodness of the SEM, model fit was evaluated using multiple indices, including the Root Mean Square Error of Approximation (RMSEA), Tucker-Lewis Index (TLI), Comparative Fit Index (CFI), and Standardized Root Mean Square Residual (SRMR). The model demonstrated a good fit, with RMSEA = 0.049, TLI = 0.92, CFI = 0.93, and SRMR = 0.03, all of which fall within widely accepted thresholds. Collectively, these indicators suggest that the model provides a strong representation of the observed data.

**Table 4:** Tests of direct and indirect effects (*n* = 257)

Effects	β	*P*-values	Percentile 95% CI Lower/Upper
Direct effects:			
Workplace ostracism leads to emotional exhaustion	**0.57**	**0.001** ^**^	0.456/0.666
Workplace ostracism leads to defensive silence	**0.47**	**0.001** ^**^	0.244/0.759
Workplace ostracism of deviant work behavior	**0.41**	**0.001** ^**^	0.235/0.540
Emotional exhaustion leads to defensive silence	**2.14**	**0.001** ^**^	1.770/2.439
Emotional exhaustion to deviant work behavior	**1.10**	**0.001** ^**^	0.937/1.289
Defensive silence to deviant work behavior	**0.07**	**0.041** ^*^	0.003/0.158
Indirect effects:			
Workplace ostracism of deviant work behavior via emotional exhaustion	**0.627**	**0.001** ^**^	0.503/0.739
Workplace ostracism of deviant work behavior via defensive silence	**0.033**	**0.041** ^*^	0.001/0.098
Workplace ostracism to deviant work behavior via emotional exhaustion and defensive silence	0.660	0.001^**^	0.516/0.788

CI: Confidence interval. * Statistically significant level at *p* < 0.05. ** Statistically significant level at *p* < 0.01

## Discussion

The nursing sector is an important aspect of Egypt’s healthcare system, in which nurses play an important role in guaranteeing patients’ well-being and providing high-quality patient care. Nevertheless, workplace deviance can take the form of tardiness, stealing, absenteeism, and verbal abuse, which directly affects an organization, resulting in lower productivity and commitment, and greater employee turnover [18]. Nurses who encounter such deviant behaviors at the workplace are more likely to have lower job motivation, quit, and feel stress on the job, which will eventually lead to low self-esteem, psychiatric disorders, and greater fear of downsizing [50]. So, this study explained the association between workplace ostracism and nurses’ deviant work behaviors, highlighting the mediating roles of emotional exhaustion and defensive silence in this relationship.

### Workplace ostracism and deviant work behaviors among nurses

The current study’s findings revealed that there was a significant and positive association between workplace ostracism and deviant work behavior. This suggests that when nurses experience exclusion, isolation, or social neglect in the workplace, they are more likely to engage in behaviors that go against professional and organizational norms. This result might be explained by the fact that when nurses feel ignored or excluded by their colleagues or supervisors, they may lose motivation, leading to reduced commitment and a decline in ethical workplace behavior. This can manifest in various forms of deviant work behavior, such as neglecting responsibilities, being uncooperative, or showing hostility toward coworkers.

The previous finding aligns with the findings of Shafique et al. [8], who investigated the impact of workplace ostracism on deviant behavior among nurses in public sector hospitals, discovered that ostracism positively correlates with deviant behavior. Correspondingly, Ahmed and Mahmoud [2] identified a positive association between workplace ostracism and counterproductive work behaviors. Similarly, Preena [51], Luo et al. [9], and Endratno et al. [52] found that workplace ostracism has a significant positive impact on employees’ deviant behavior. Conversely, Gharaei et al. [14] found that nurses’ experiences of workplace ostracism were significantly related to factors such as employment status and perceptions of managerial discrimination. However, it did not establish a direct link between ostracism and deviant work behaviors, suggesting that other variables may mediate this relationship.

### Workplace ostracism and emotional exhaustion among nurses

The current study findings discovered a significant and positive association between workplace ostracism and emotional exhaustion. This outcome may be attributed to the fact that workplace ostracism, characterized by exclusion, social isolation, and a lack of support, can be highly distressing for employees. Consequently, nurses who experience ostracism may feel undervalued, disconnected, and unsupported in their professional environment, leading to increased psychological strain. The continuous stress of being ignored or excluded can deplete emotional resources, resulting in emotional exhaustion. This result is in the same line with the results of Qi et al. [24], Chen and Li [53], and Endratno et al. [52], who indicated that workplace ostracism is strongly and positively correlated with emotional exhaustion.

### Workplace ostracism and defensive silence among nurses

The findings of the current study revealed a significant and positive relationship between workplace ostracism and defensive silence. This result may be explained by the idea that workplace ostracism, which involves social exclusion, ignoring, or marginalizing an individual, can create an environment of fear, insecurity, and distrust. Nurses who experience ostracism may choose to withhold their opinions, concerns, or suggestions as a self-protective strategy, fearing further exclusion, negative judgment, or retaliation. Additionally, in healthcare settings, where teamwork and communication are critical, nurses who feel excluded may become hesitant to express their viewpoints, report errors, or share innovative ideas, leading to a decline in collaboration and patient care quality. The psychological distress caused by ostracism fosters a perception that voicing concerns could lead to further isolation, reinforcing a cycle of silence.

The previous finding aligns with the results of Saifa et al. [54], Elhanafy and Ebrahim [15], Özişli [55], Yao et al. [56], and Ismail et al. [57], who stated a strong and positive correlation between workplace ostracism and defensive silence. On the other hand, Khalid et al. [58] reported that the relationship between workplace ostracism and defensive silence is not statistically significant.

### The mediating effect of emotional exhaustion

The current study findings demonstrated that the relationship between workplace ostracism and deviant work behavior among staff nurses was mediated by emotional exhaustion. This is explained by the fact that workplace ostracism generates a stressful environment in which nurses feel undervalued, isolated, and unsupported. This prolonged psychological distress depletes emotional resources, leading to emotional exhaustion. As emotional exhaustion intensifies, nurses may struggle to regulate their emotions, leading to frustration, disengagement, and an increased likelihood of engaging in deviant work behaviors, such as negligence, withdrawal, or counterproductive workplace actions.

This result aligns with that of Jiang et al. [7] and Kemal et al. [39], which indicated that the association between workplace ostracism and deviant behavior was mediated by emotional exhaustion. Congruently, Qi et al. [24] demonstrated that emotional exhaustion serves as a mediator in the relationship between workplace ostracism and unethical behavior. Harmoniously, Saifa et al. [54] reported that the direct link between workplace ostracism and counterproductive work behavior is more comprehensively understood when considering emotional exhaustion as a mediating factor.

### The mediating effect of defensive silence

The present study revealed that defensive silence significantly mediates the association between workplace exclusion and deviant work behavior. This could be because when nurses feel excluded, they may withhold their thoughts and concerns as a form of self-protection strategy in response to ostracism. However, this silence can contribute to frustration and resentment, ultimately leading to deviant behaviors, such as taking longer breaks or even sabotaging the organization. This finding is consistent with Jahanzeb and Fatima [6], who identified defensive silence as a key mediator in the association between workplace ostracism and deviant behavior. Similarly, Saifa et al. [54] highlighted that the direct association between workplace ostracism and counterproductive work behavior becomes clearer when defensive silence is taken into account as a mediating variable.

### The serially mediating effects of emotional exhaustion and defensive silence

Regarding the final research hypothesis, the current findings suggest that the association between workplace ostracism and deviant behavior is serially mediated by emotional exhaustion and defensive silence. This could be because nurses who experience ostracism in the workplace are deprived of social support and may feel isolated and undervalued. As a result, this depletes the important psychological and emotional resources among nurses and eventually causes feelings of overstretching or emotional exhaustion. Accordingly, nurses become mute observers and adopt defensive silence as a self-protection policy. By withholding their ideas or concerns, they aim to shield themselves from further emotional depletion and avoid conflicts that could exacerbate their exhaustion. When nurses feel they cannot speak up, they attempt to distance themselves from job demands and participate in retaliatory deviant behaviors.

In the same way, Jahanzeb and Fatima [6] revealed that defensive silence and emotional exhaustion act as sequential mediators in the relationship between workplace ostracism and interpersonal deviance. Likewise, Saifa et al. [54] emphasized that the direct link between workplace ostracism and counterproductive work behavior is better understood when considering defensive silence and emotional exhaustion as mediating factors.

### Strengths and limitations

This study expands existing research by integrating emotional exhaustion and defensive silence as mediators in the relationship between workplace ostracism and deviant work behavior, offering a deeper understanding of the underlying psychological mechanisms. Findings provide actionable insights for healthcare organizations, emphasizing the need for inclusive workplace policies to minimize ostracism and its negative consequences on nurses and patient care. Additionally, by examining nurses, a profession heavily reliant on teamwork and communication, the study addresses a critical issue in the healthcare sector, where workplace dynamics directly impact service quality and patient outcomes. Moreover, the study uses quantitative methods, ensuring data-driven conclusions that strengthen the reliability of its findings.

However, there are certain limitations of the study as follows: The study relies on self-reported data, which may be subject to social desirability bias or response distortion, as participants might underreport deviant behaviors or overstate positive actions to present themselves favorably. Additionally, the study was conducted in a specific geographic and organizational context, potentially limiting the generalizability of findings to nurses in different healthcare settings, cultural contexts, or regions. Furthermore, other potential mediators (e.g., personality traits, organizational culture, or leadership styles) may also influence the relationship between workplace ostracism and deviant work behavior but were not included in this study.

### Implications for nursing management and leadership

Addressing workplace ostracism is essential for nursing management to mitigate its negative impact on nurses’ well-being and organizational outcomes. To effectively tackle this issue, nursing managers can implement the following actionable strategies:​ (1) Create clear policies that define workplace ostracism and outline procedures for reporting and addressing such behaviors.​ (2) Ensure all staff is educated about these policies and the importance of fostering an inclusive work environment. (3) Set up anonymous channels for nurses to report incidents of ostracism without fear of retaliation.​ (4) Regularly monitor and address reports to demonstrate a commitment to a supportive workplace culture.​ (5) Offer workshops focused on communication, empathy, and teamwork to enhance staff interactions and reduce misunderstandings.​.

6) Include training on recognizing and addressing ostracism and its consequences.​ 7) Encourage regular team meetings where nurses can voice concerns, share ideas, and discuss challenges.​ 8) Foster a culture where feedback is welcomed and valued, reducing the likelihood of defensive silence.​ 9) Assess and adjust workloads to ensure they are manageable, thereby reducing stress and emotional exhaustion among staff.​ 10) Provide resources and support for stress management and resilience-building.​ 11) Acknowledge and reward behaviors that contribute to a positive and inclusive work environment.​ 12) Celebrate teamwork and collaborative achievements to reinforce a sense of belonging.​ 13) Implement surveys and assessments to gauge the prevalence of ostracism and its impact on staff.​ 14) Use the findings to inform policy adjustments and targeted interventions.

## Conclusion

This study provides significant insights into the relationship between workplace ostracism and deviant work behaviors among nurses, emphasizing the mediating roles of emotional exhaustion and defensive silence. Findings suggest that workplace ostracism is a critical workplace stressor that leads to psychological strain and contributes to deviant work behaviors among nurses. When nurses experience social exclusion, they may suffer from emotional exhaustion due to a lack of social support, making it difficult to remain engaged and motivated. This emotional depletion increases the likelihood of engaging in deviant work behaviors as a maladaptive coping mechanism. Additionally, ostracized nurses may resort to defensive silence by withholding their opinions, concerns, or vital work-related information to protect themselves from further exclusion or negative repercussions, which further contributes to workplace deviance.

These findings underscore the importance of fostering inclusive work environments where nurses feel valued and supported. Healthcare organizations should implement policies that promote social belonging, psychological safety, and open communication to reduce the adverse effects of workplace ostracism. By addressing emotional exhaustion and encouraging constructive dialogue, organizations can mitigate deviant work behaviors and improve overall workplace morale, ultimately protect nurse well-being, ensure safer, higher-quality patient care, and organizational effectiveness.

## Data Availability

The data supporting the findings of this study are available from the corresponding authors upon reasonable request. Furthermore, all data generated or analyzed during this research are included in this publication.

## Abbreviations

EES: Emotional Exhaustion Scale
WDQ: Workplace Deviance Questionnaire
AMOS: Analysis of Moment Structures
SEM: Structural Equation Modeling
ANOVA: Analysis of Variance
RMSEA: Mean Square Error of Approximation
TLI: Tucker-Lewis Index
CFI: Comparative Fit Index
SRMR: Standardized Root Mean Square Residual
